# Therapeutics targeting CD90-integrin-AMPK-CD133 signal axis in liver cancer

**DOI:** 10.18632/oncotarget.5976

**Published:** 2015-10-22

**Authors:** Wei-Ching Chen, Yung-Sheng Chang, Hui-Ping Hsu, Meng-Chi Yen, Hau-Lun Huang, Chien-Yu Cho, Chih-Yang Wang, Tzu-Yang Weng, Po-Ting Lai, Ching-Shih Chen, Yih-Jyh Lin, Ming-Derg Lai

**Affiliations:** ^1^ Department of Biochemistry and Molecular Biology, College of Medicine, National Cheng Kung University, Tainan, Taiwan; ^2^ Institute of Basic Medical Sciences, College of Medicine, National Cheng Kung University, Tainan, Taiwan; ^3^ Department of Surgery, College of Medicine, National Cheng Kung University, Tainan, Taiwan; ^4^ Division of Medicinal Chemistry, College of Pharmacy, The Ohio State University, Columbus, OH, USA; ^5^ Center for Infectious Diseases and Signaling Research, College of Medicine, National Cheng Kung University, Tainan, Taiwan

**Keywords:** cancer stem cell marker, integrin, AMPK, mTOR, OSU-CG5

## Abstract

CD90 is used as a marker for cancer stem cell in liver cancer. We aimed to study the mechanism by which CD90 promoted liver cancer progression and identify the new therapeutic targets on CD90 signal pathway. Ectopic expression of CD90 in liver cancer cell lines enhanced anchorage-independent growth and tumor progression. Furthermore, CD90 promoted sphere formation *in vitro* and upregulated the expression of the cancer stem cell marker CD133. The CD133 expression was higher in CD45-CD90+ cells in liver cancer specimen. The natural carcinogenic molecules TGF-β-1, HGF, and hepatitis B surface antigen increased the expression of CD90 and CD133. Inhibition of CD90 by either shRNA or antibody attenuated the induction of CD133 and anchorage-independent growth. Lentiviral delivery of CD133 shRNA abolished the tumorigenicity induced by CD90. Ectopic expression of CD90 induced mTOR phosphorylation and AMPK dephosphorylation. Mutation of integrin binding-RLD domain in CD90 attenuated the induction of CD133 and anchorage-independent growth. Similar results were observed after silencing β3 integrin. Signaling analyses revealed that AMPK/mTOR and β3 integrin were required for the induction of CD133 and tumor formation by CD90. Importantly, the energy restriction mimetic agent OSU-CG5 reduced the CD90 population in fresh liver tumor sample and repressed the tumor growth. In contrast, sorafenib did not decrease the CD90+ population. In conclusion, the signal axis of CD90-integrin-mTOR/AMPK-CD133 is critical for promoting liver carcinogenesis. Molecules inhibiting the signal axis, including OSU-CG5 and other inhibitors, may serve as potential novel cancer therapeutic targets in liver cancer.

## INTRODUCTION

Liver cancer is a common cause of cancer-related deaths around the world and is a result of the accumulation of genetic and epigenetic alterations. There are several etiological factors and potent stimulators involved in liver cancer progression, such as hepatitis B virus (HBV), transforming growth factor-β-1 (TGF-β-1), and hepatocyte growth factor (HGF). The upregulation of TGF-β-1 in hepatocellular carcinoma (HCC) correlates with hepatic carcinogenesis and tumor progression [1]. The serum levels of HGF are elevated in a variety of liver diseases [2], and HGF concentrations are used as a tumor marker for HCC [3]. In addition, the hepatitis B virus surface antigen is shown to promote tumor formation [4]. A mutated HBV large surface protein with a deletion in the antigen region has been found in the serum of patients with HBV infection, and this protein enhances anchorage-independent growth [5]. The etiologic agents have been proposed to induce tumor progression through the hierarchy model or the stochastic model (these models are not mutually exclusive). In the stochastic model, the instigating cells acquire a proliferative advantage over the surrounding cells through the accumulation of multiple genetic and epigenetic alterations, as demonstrated in the development of colon cancer [6]. Alternatively, tumors may develop according to the hierarchical model, which proposes that a small population of cells within each tumor has the ability to generate a tumor and recapitulate the traits of a whole tumor [7]. These tumor-initiating cells are called cancer stem cells (CSCs). CSCs generate tumors through the stem cell processes of self-renewal and differentiation into multiple cell types; this hypothesis has been supported by studies in which a small population of leukemia cells had the ability to recapitulate the whole tumor [8].

To identify cancer-initiating cells, scientists generally separate the tumor cells into various subpopulations using cell surface markers and examine their tumor-forming ability in immunocompromised mice [9, 10]. Several representative cell surface markers have been identified from human hepatocarcinoma cell lines and primary tissues [11, 12]. These hepatocarcinoma CSC markers include CD133, CD90, CD44, CD24, EpCAM, and OV6. CD133, also called prominin 1, is a 5-transmembrane protein and a marker of normal hematopoietic stem cells. Injection of as few as 100 CD133+ brain tumor cells was found to form tumors in xenotransplantation assays, whereas the same number of CD133-cells was unable to generate tumors [13]. In addition to brain cancer, CD133 was later used to purify CSCs from several other tumor types [14, 15]. Expression of CD133 enhances malignancy by matrix metalloproteinase (MMP)-2 and a disintegrin and metalloproteinase (ADAM) 9. CD133 increases the colony-formation ability and alters the cell cycle in HCC [16]. The increased CD133 expression correlates with poor prognosis in HCC [17]. CD133-positive HCC cells possess a greater ability to grow in soft agar and to form tumors *in vivo* than the corresponding CD133-negative cells [18, 19]. The expression of CD133 is regulated by DNA methylation. TGF-β-1 induces CD133 expression through the inhibition of DNMT1 and DNMT3β, and this inhibition is partially dependent on the SMAD pathway [20]. Yang et al. identify CSCs from HCC cell lines and primary HCC tissues that are defined by the expression of the hepatic progenitor marker OV6 and activation of Wnt/β-catenin signaling [21]. Gene expression and signaling pathway analyses on HCC specimens reveal that cells positive for the surface hepatic stem cell marker EpCAM have features of cancer stem cells [22]. Because some CD133+ cells are representative of CSCs, further identification and characterization reveal that CSCs could be better defined by co-expression of CD133 and CD44 on the cell surface [23]. In contrast, the number of cells expressing CD90 (Thy1), a glycosylphosphatidylinositol (GPI)-anchored glycoprotein, is correlated with the tumorigenicity of HCC cell lines. The CD90+CD44+ cells possess a more aggressive and metastatic phenotype than the CD90+CD44− cells [24]. The function of CD90 may be dependent on cell type; activation of CD90 induces the activation and translocation of FasL via the src family kinases in lung myofibroblasts [25]. A decrease in CD90 expression has been observed in nasopharyngeal cell lines and in 65% of tumor samples. Restoration of CD90 expression causes a decrease in colony formation [26]. CD90 has an RGD-like sequence, RLD, and it binds to ανβ3 integrin through its RLD sequence, thereby activating the interaction between melanoma cells and activated endothelial cells [27–29]. The binding of CD90 to ανβ5 integrin is RLD-dependent because the mutated form, CD90-RLE, loses the ability to bind to the integrin on lung fibroblasts. Furthermore, the liver cancer stem cells have been classified as two groups with EpCAM or CD90 [30].

Targeted therapy is one type of cancer treatment that uses drugs to more precisely attack cancer cells. The drug development for targeted therapy is usually based on the specific mutation or dysregulated signaling pathway in cancer. Several signaling pathways, including the MAPK/ERK, PI3K/AKT/mTOR, STAT3, VEGFR and PDGFR pathway, are demonstrated to promote cancer progression [31, 32]. Sorafenib inactivates ERK and mTOR signaling pathway and suppresses the tumor formation [33]. The combination of sorafenib and PKI-587 drives the inhibition of proliferation in liver cancer [34]. Recently, studies have indicated that cancer-initiating cells may benefit from the abundant expression of CD44 [35]. Cancer stem cell marker is not only used to define specific populations of cancer cells, but also correlates with tumor growth. Therefore, we aimed to study whether CD90 CSC marker and its downstream signaling pathway play an important role in tumor growth. In this report, we demonstrate that abundantly expressed CD90 increases sphere formation, soft agar growth, and tumorigenicity in HepG2 and Hep3B cells. In addition, CD90 enhances the expression of CD133 via the AMPK and mTOR pathways. The binding of CD90 to integrin through the RLD residues is essential for the induction of CD133 and soft agar growth. The reduction in CD133 expression attenuates the induction of soft agar growth by CD90. Our results demonstrate that the CD90-integrin-AMPK-CD133 signal axis is essential for the growth of liver cancer. Therapeutics targeting the signal axis may be useful for liver cancer treatment.

## RESULTS

### CD90 promotes tumorigenicity in HepG2, Hep3B and HuH7 cells

**Figure 1:**
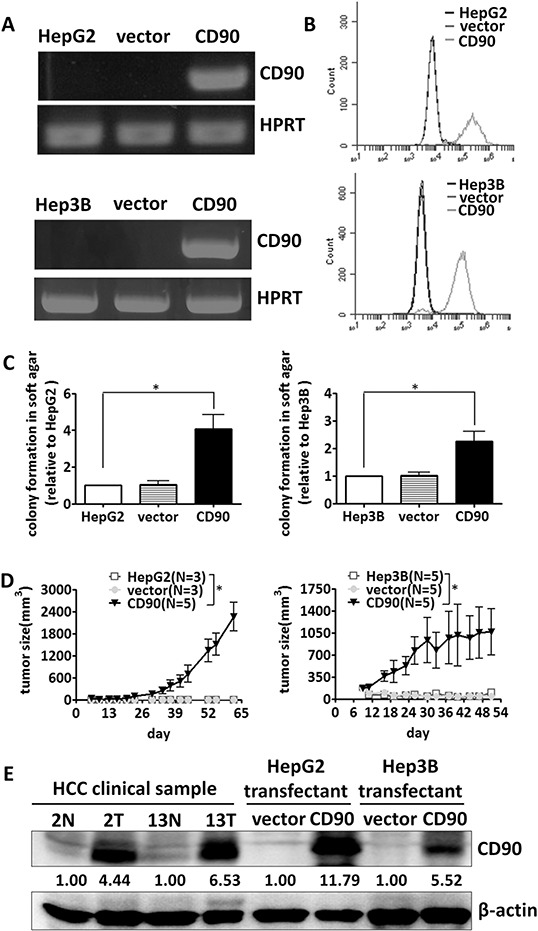
CD90 promotes tumor formation in liver cancer cells CD90 mRNA expression and cell surface expression in the exogenous CD90-overexpressing stable transfectants were determined by RT-PCR **A.** and FACS analyses **B. C.** The transfectants were plated in soft agar, and the colonies were monitored for 14 days. The colonies were quantified using Image-Pro Plus software. Data represent mean ± SEM (*n* = 3). *P* value was calculated using one-way anova analysis and * indicated *P* < 0.01. **D.** The transfected cells were injected subcutaneously into NOD/SCID mice, and tumor growth curves were determined. Data represent mean ± SEM. *P* value was calculated using two-way anova analysis and * indicated *P* < 0.0001. **E.** The protein expression of CD90 in the transfectants and two pairs of tumor samples was analyzed by western blotting with an anti-CD90 antibody.

To determine whether the CD90 cancer stem cell marker is involved in the tumorigenesis of liver cancer cell, HepG2, Hep3B and HuH7 cells were transfected with a plasmid encoding CD90. Ectopic expression of CD90 mRNA was detected by RT-PCR analysis, and surface expression of CD90 was verified by flow cytometry analysis (Figure 1A and 1B, Supplementary Figure S1A and S1B). Ectopic expression of CD90 increased anchorage-independent growth *in vitro* and tumor formation *in vivo* (Figure 1C and 1D, Supplementary Figure S1C and S1D). Furthermore, the expression of CD90 in the stable transfectants was comparable to the expression of CD90 in the liver tumor samples, suggesting that the phenotype was not due to artificial overexpression in cell lines (Figure 1E).

### CD90 increases sphere-forming ability and the expression of stem cell marker CD133

**Figure 2:**
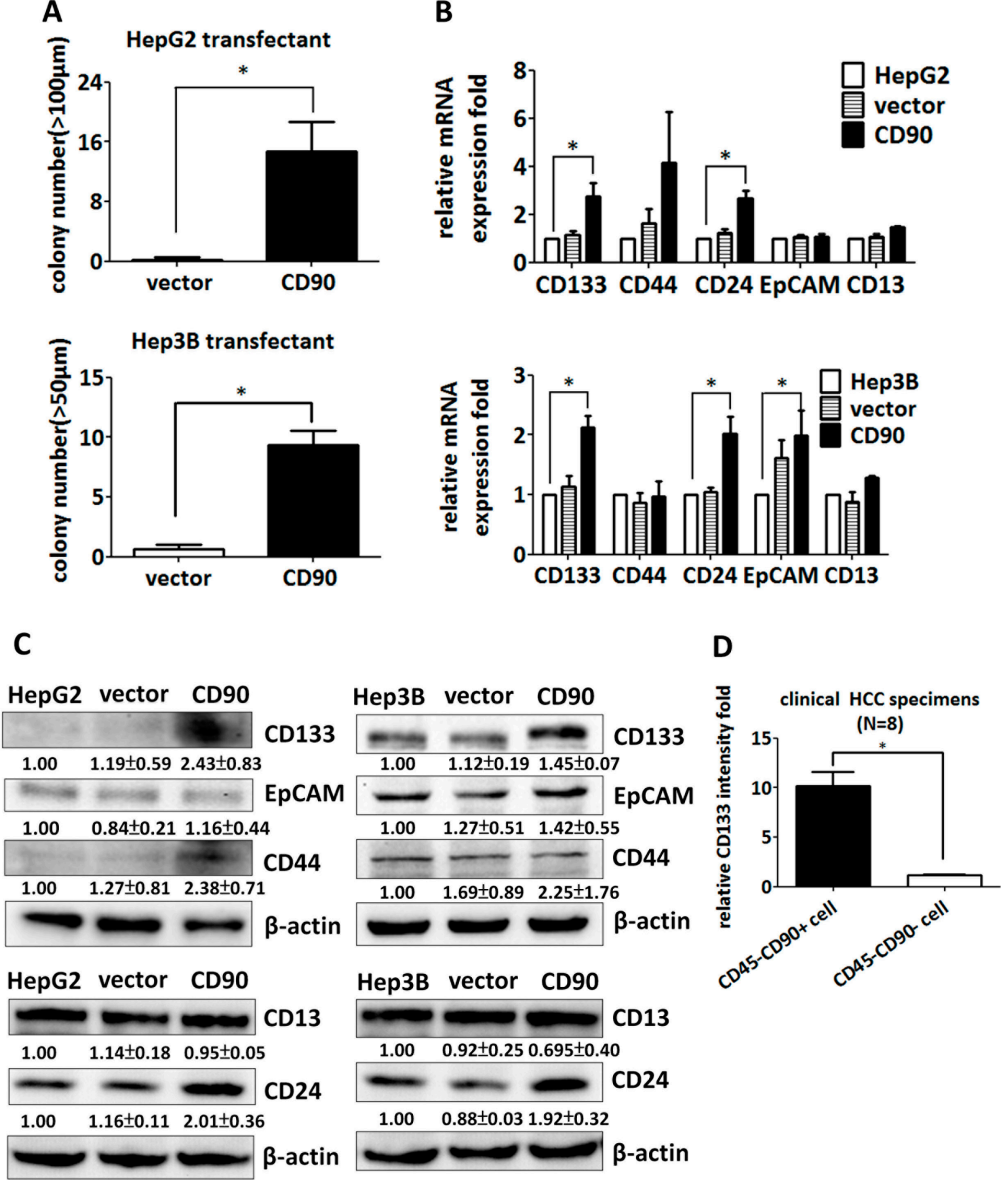
CD90 increases sphere formation and the expression of cancer stem cell markers in cell lines and tumor specimens **A.** The transfectants were seeded into ultra-low attachment plates, and the numbers of formed spheres were counted. Data represent mean ± SEM (*n* = 4). *P* value was calculated using unpaired *t*-test and * indicated *P* < 0.01. **B.** The expression of the liver cancer stem cell markers CD133, CD44, CD24, EpCAM, and CD13 was determined by quantitative RT-PCR in CD90 transfectants, control transfectants, and parental cells. The mRNA levels were normalized to HPRT. Data represent mean ± SEM (*n* = 3). *P* value was calculated using one-way anova analysis and * indicated *P* < 0.05. **C.** The expression of CD133, CD44, EpCAM, CD24, and CD13 in the transfectants was determined by western blotting. The quantitative data represent mean ± SD (*n* = 3). **D.** The CD133 expression of the CD45-CD90+ and CD45-CD90- populations in freshly isolated HCC specimens was determined by flow cytometry. The quantitative data represent mean ± SEM (*n* = 8). *P* value was calculated using unpaired *t*-test analysis and * indicated *P* < 0.0001.

Ectopic expression of CD90 enhanced sphere formation in ultra-low attachment culture dishes, a capability that is one of the characteristics of cancer stem cells (Figure 2A). The expression of CD133 was dramatically enhanced by the ectopic expression of CD90 (Figure 2B). The expression of other stem cell markers, including CD44, EpCAM, and CD13, was not statistically different between the HepG2 CD90 transfectants and the parental cells (Figure 2B). In contrast, the expression of CD24 was higher in the CD90 transfectants (Figure 2B). The elevated expression of CD133 was further confirmed by western blotting (Figure 2C). Taken together, the results indicate that the expression of CD133 and CD24 are induced by the ectopic expression of CD90. CD133 is a CSC marker in many types of cancer, implicating its significance of cancer development. To confirm the upregulation of CD133 *in vivo*, we analyzed the expression of CD133 in tumor cells from clinical HCC samples. The CD45- population was used to exclude the effects from CD45+ cells. The expression of CD133 in the CD45-CD90+ tumor cells was higher than that in the CD45-CD90- tumor cells isolated from clinical HCC specimens (Figure 2D).

### Inactivation of CD90 by either shRNA or antibody inhibits anchorage-independent growth and CD133 expression which are upregulated by environmental stimuli

**Figure 3:**
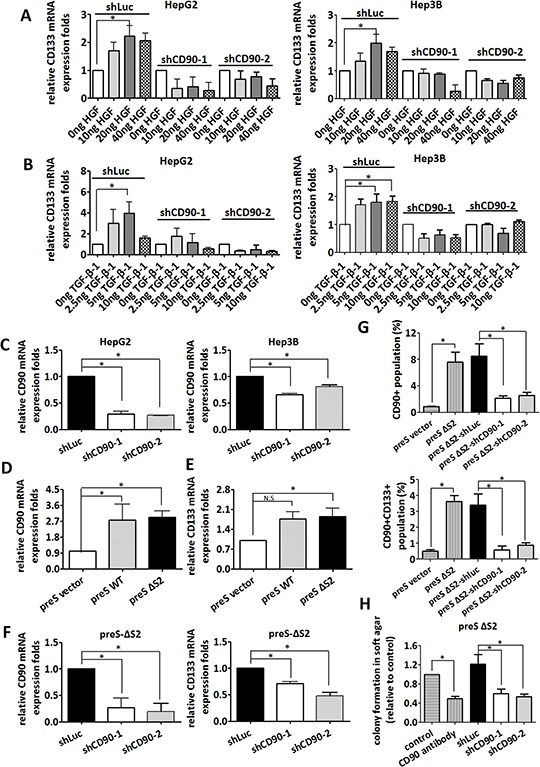
Silencing of CD90 inhibits anchorage-independent growth and CD133 expression induced by environmental stimuli including HGF, TGF-β-1, or HBV large surface protein HepG2 and Hep3B cells were infected with lentiviral particles containing shCD90 or shLuc. The infected HepG2 and Hep3B cells were then treated with HGF **A.** and TGF-β-1 **B.** at the indicated doses for 24 hours. CD133 mRNA levels were then determined by quantitative RT-PCR. **C.** The downregulation of CD90 mRNA was determined by quantitative RT-PCR. Data represent mean ± SEM (*n* = 3). **D.** The expression of CD90 in liver cancer HuH7 cells expressing the PreS2 deletion (PreS-ΔS2) mutant HBV large surface protein was examined by quantitative RT-PCR. Data represent mean ± SEM (*n* = 4). **E.** The expression of CD133 in liver cancer HuH7 cells expressing the PreS2 deletion (PreS-ΔS2)) mutant HBV large surface protein was examined by quantitative RT-PCR. Data represent mean ± SEM (*n* = 5). **F.** After infection with lentiviral particles containing shCD90 and shLuc, total mRNA from the PreS-ΔS2 transfectants was extracted, and the expression of CD90 and CD133 was examined by quantitative RT-PCR. Data represent mean ± SEM (*n* = 4). **G.** After infection with lentiviral particles containing shCD90 and shLuc, the surface CD90 and CD133 in liver cancer HuH7 cells expressing the PreS2 deletion (PreS-ΔS2) mutant HBV large surface protein was examined by flow cytometry. Data represent mean ± SEM (*n* = 4). **H.** Anchorage-independent growth was determined using soft agar assay after lentiviral infection of the indicated shRNA or CD90 antibody treatment. Data represent mean ± SEM (*n* = 3). All *P* value was calculated using one-way anova analysis and * indicated *P* < 0.05.

HGF and TGF-β-1 promotes liver cancer progression, and the serum levels of HGF and TGF-β-1 are elevated in hepatic carcinogenesis [1–3]. HGF and TGF-β-1 induced the expression of CD133 in HepG2 and Hep3B cells, and the expression of CD133 was significantly reduced by the introduction of CD90 shRNA (Figure 3A and 3B). The knockdown efficacy induced by CD90 shRNA in HepG2 and Hep3B cell was analyzed using quantitative RT-PCR (Figure 3C). Our previous report has indicated that HBV pre-S2 large surface protein induces a variety of pathologic conditions in the hepatocarcinoma HuH7 cell [5]. The pre-S2 mutant gene with a deletion of pre-S2 region of large surface protein (ΔS2-LHBs) contains a deletion at nucleotides 4–57. ΔS2-LHBs is clustered in the hepatocyte with hepatitis B virus infection and involved in the tumorigenesis. The expression of CD133 and CD90 in HuH7 transfectants expressing wild-type LHBs and ΔS2-LHBs was analyzed by quantitative RT-PCR (Figure 3D and 3E). Furthermore, the induction of CD133 by the expression of the HBV large surface protein was attenuated by CD90 shRNA (Figure 3F and 3G). Finally, anchorage-independent growth of HBV-preS2 cells was abolished by shRNA or antibody against CD90 (Figure 3H). To further confirm the effect of CD90 shRNA in liver cancer, the lentiviral particles containing CD90 shRNA was delivered to liver cancer PLC/PRF/5 cells. The CD133 expression and anchorage-independent growth in the PLC/PRF/5 cell were attenuated by CD90 shRNA (Supplementary Figure S2A and S2B). Taken together, the results indicate that CD90 is essential for the induction of CD133 by physiological stimuli (HGF and TGF-β-1) or pathological alteration (the HBV large surface protein).

### Inhibition of CD133 abolishes the tumor growth induced by CD90

**Figure 4:**
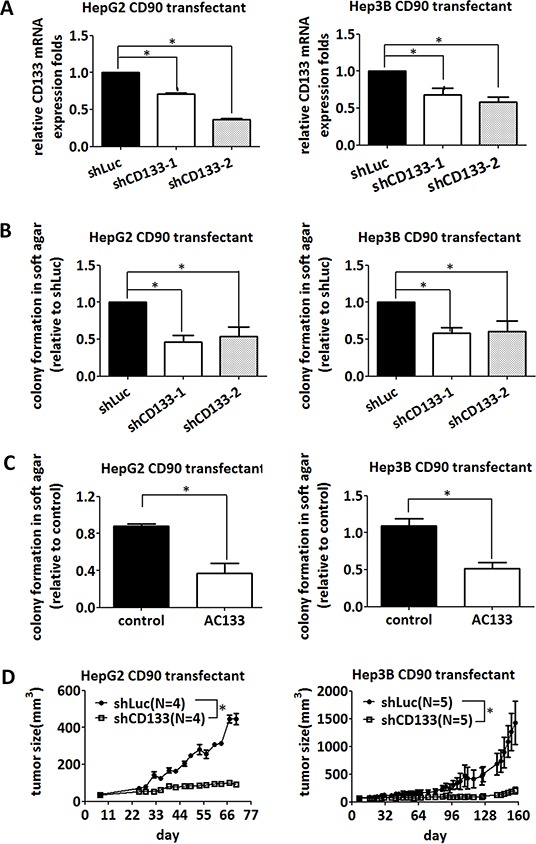
Inhibition of CD133 attenuates the anchorage-independent growth induced by CD90 **A.** CD90 transfectants were infected with two different shRNAs targeting CD133, and the downregulation of CD133 mRNA was determined by quantitative RT-PCR. Data represent mean ± SEM (*n* = 4). **B.** CD90-transfected HepG2 and Hep3B cells were infected with two different shRNAs targeting CD133, and the anchorage-independent growth was determined in soft agar. Data represent mean ± SEM (*n* = 4). *P* value was calculated using one-way anova analysis and * indicated *P* < 0.05. **C.** CD90-transfected HepG2 and Hep3B cells were seeded in soft agar and incubated with the anti-CD133 antibody (AC133). Colonies were counted after 14 days. Data represent mean ± SEM (*n* = 3). *P* value was calculated using unpaired *t*-test analysis and * indicated *P* < 0.05. **D.** CD90 transfectants were subcutaneously co-injected with lentiviral particles containing luciferase or CD133 shRNA into NOD/SCID mice. The size of tumor was measured twice a week. *P* value was calculated using two-way anova analysis and * indicated *P* < 0.05.

Since overexpression of CD90 induced the expression of CD133 and anchorage-independent growth, we investigated whether CD133 was essential for the anchorage-independent growth induced by CD90. Lentiviral introduction of CD133 shRNA was used to inhibit CD133 expression (Figure 4A). Two different CD133 shRNAs significantly suppressed the anchorage-independent growth induced by CD90 in HepG2 and Hep3B CD90 transfectants (Figure 4B). Treatment with a monoclonal anti-CD133 antibody (AC133) also blocked the anchorage-independent growth induced by CD90 in hepatocellular carcinoma cells (Figure 4C), excluding the off-target effects of CD133 shRNA in general. Finally, lentiviral introduction of shCD133 *in vivo* inhibited tumor growth (Figure 4D). Thus, CD133 is required for the CD90-induced tumor progression.

### CD90-AMPK/mTOR-CD133 signaling axis functions as therapeutic target

**Figure 5:**
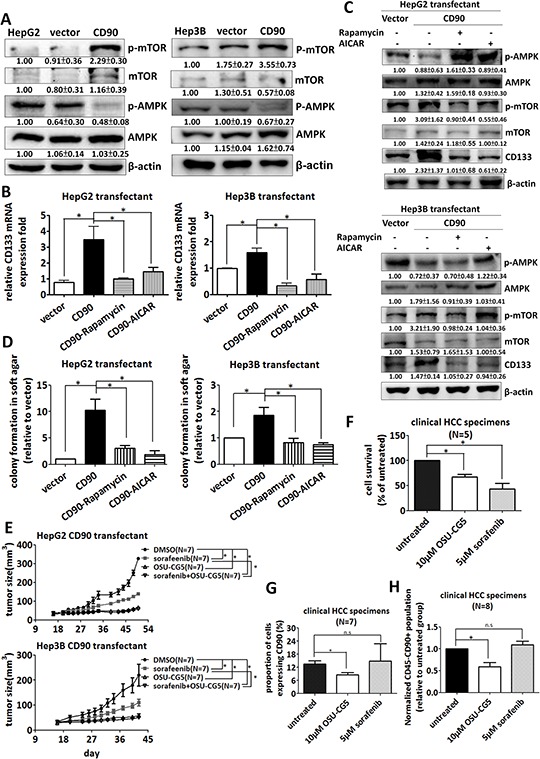
Inhibition of the AMPK/mTOR pathway attenuates the upregulation of CD133 by CD90 in cell lines and fresh liver cancer specimen **A.** The phosphorylation of mTOR and AMPK in the transfectants was determined by western blotting. The quantitative data represent mean ± SD (*n* = 3). CD133 mRNA **B.** and protein expression **C.** in the transfectants were determined by quantitative RT-PCR and western blotting respectively after rapamycin or AICAR treatment for 24 hours. **D.** The transfectants were plated in soft agar and treated with the indicated inhibitors. Colonies were monitored for 14 days and quantified using Image-Pro Plus software. Data represent mean ± SEM (*n* = 3). **E.** The CD90 transfectant was subcutaneously injected to NOD/SCID mice. The mice were intraperitoneally injected with indicated sorafenib or OSU-CG5 after 14 days and the drug was administrated to mice every other day. *P* value was calculated using two-way anova analysis and * indicated *P* < 0.0001. **F, G.** and **H.** The liver cancer cells from the freshly isolated HCC specimens were incubated with OSU-CG5 or sorafenib for 24 hours. The cell numbers were counted in (F), and the CD90 expression was determined by flow cytometry in (G) The population of CD45-CD90+ cancer stem cell-like was determined by flow cytometry in (H) Data represent mean ± SEM. *P* value was calculated using one-way anova analysis and * indicated *P* < 0.01.

The mTOR and AMPK pathways are involved in the maintenance of cancer stem cells [37]; thus, we investigated whether the mTOR and AMPK pathways contributes to the expression of CD133 induced by ectopic expression of CD90. HepG2 and Hep3B CD90 transfectants had higher levels of mTOR phosphorylation and lower levels of AMPK phosphorylation than the parental cells or control transfectants (Figure 5A). In contrast, the PLC/PRF/5 cells infected with lentiviral particles containing CD90 shRNA had lower levels of mTOR phosphorylation and higher levels of AMPK phosphorylation (Supplementary Figure S2C). To further determine whether mTOR and AMPK were essential for the upregulation of CD133 by CD90, we determined the effects of rapamycin (an mTOR inhibitor) and AICAR (an AMPK activator) on the expression of CD133 in the CD90 transfectants. CD133 mRNA and protein upregulation were attenuated upon rapamycin or AICAR treatment in HepG2 and Hep3B CD90 transfectants (Figure 5B and 5C). Furthermore, the increase of the anchorage-independent growth in CD90 transfectants was restored by rapamycin or AICAR (Figure 5D). An energy restriction mimetic agent OSU-CG5 has been demonstrated to inhibit mTOR pathway [38, 39]. We therefore examined the effect of OSU-CG5 on the CD90-induced anchorage-independent growth. The OSU-CG5 selectively inhibited the growth and the mTOR phosphorylation of HepG2 CD90 transfectants and Hep3B CD90 transfectants (Supplementary Figure S3A). OSU-CG5 had a comparable killing effect with sorafenib on the parental HepG2 and Hep3B cells (Supplementary Figure S3B), and selectively reduced CD90 population in Hep3B cells (Supplementary Figure S3C). Importantly, OSU-CG5 alone significantly abolished tumor growth in animal tumor model, but did not grossly decrease the weight for mice, an indication for health condition (Figure 5E and Supplementary Figure S3D). Finally, we determined whether OSU-CG5 can specifically eradicate the CD90-positive population in clinical tumor samples. OSU-CG5 had the killing effect on tumor cells and decreased the CD90-positive population in the fresh liver tumors (Figure 5F and 5G). In contrast, sorafenib, an inhibitor mainly works on ERK pathway [40], did not suppress the CD90-positive population cells (Figure 5G). Compared to the sorafenib, OSU-CG5 significantly inhibited the CD45-CD90+ populations from clinical tumor samples (Figure 5H). Altogether, the CD90-AMPK/mTOR-CD133 signal axis is required for hepatocarcinogenesis. OSU-CG5 was able to decrease the CD45-CD90+ tumor cells, which were potentially a population of liver cancer stem cells.

### The CD90 RLD domain affects anchorage-independent growth and CD133 expression

CD90 has been shown to promote lung fibroblast differentiation through an interaction with integrin through its RLD sequence [41]. We sought to determine whether the RLD domain of CD90 is essential for the signal transduction in regulating CD133 expression. The RLD residue of CD90 was replaced with RLE, and the mutant CD90 was transfected into HepG2 and Hep3B cells. Neither anchorage-independent growth nor CD133 mRNA expression was induced by the CD90 RLE mutant (Supplementary Figure S4A and S4B). The alteration on AMPK phosphorylation and mTOR phosphorylation was attenuated in the cell lines expressing CD90 RLE mutant (Supplementary Figure S4C).

### Silencing of β3 Integrin decreases tumor formation and CD133 expression

**Figure 6:**
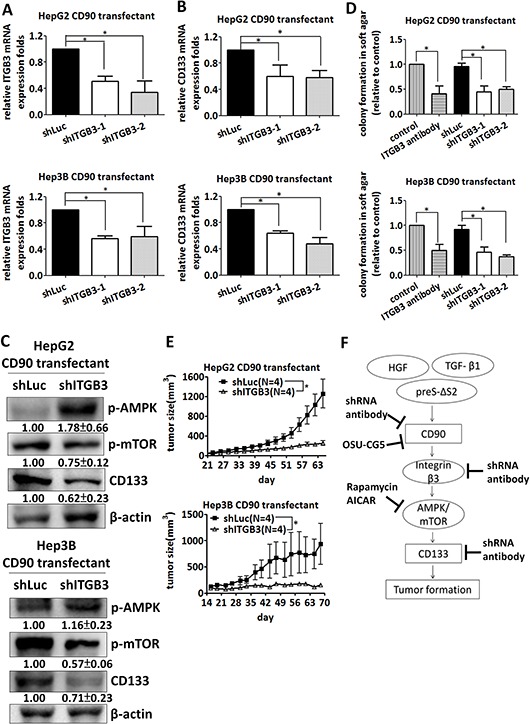
Silencing of β3 integrin inhibits tumor formation and CD133 expression induced by CD90 **A.** β3 Integrin mRNA was downregulated in CD90 transfectants with lentiviral particles containing shRNA targeting β3 integrin or luciferase. The mRNA was determined by quantitative RT-PCR. Data represent mean ± SEM (*n* = 3). **B.** CD133 mRNA expression in the cells expressing β3 integrin or luciferase shRNA was determined by quantitative RT-PCR. Data represent mean ± SEM (*n* = 5). **C.** The phosphorylation of mTOR and AMPK and the expression of CD133 were determined by western blotting. Data represent mean ± SD (*n* = 3). **D.** Anchorage-independent growth of HepG2 or Hep3B CD90 transfectant was determined using soft agar assay after lentiviral infection of the indicated shRNA or β3 integrin antibody treatment Data represent mean ± SEM (*n* = 5). *P* value was calculated using one-way anova analysis and * indicated *P* < 0.05. **E.** CD90 transfectants were co-injected with lentiviral particles containing luciferase or β3 integrin shRNA into NOD/SCID mice. The tumor growth was monitored twice a week. Data represent mean ± SEM (*n* = 4). *P* value was calculated using two-way anova analysis and * indicated *P* < 0.05. **F.** A model is presented to illustrate the signal axis of CD90 and CD133. CD90 binds to β3 integrin and increases the expression of CD133 and tumor growth. The AMPK/mTOR pathway is essential for the induction of CD133. The indicated shRNAs, antibodies and inhibitors suppress the growth of tumor cells *in vitro* and *in vivo*.

Integrins recognize arginine-glycin-aspartic acid (RGD) sequence and contribute to the cancer cell growth. The RGD-like sequence, RLD, also binds to integrin and participates in the signaling transduction. We sought to identify the effect of integrin family in CD90 transfectant. The lentiviral particles containing αV, β1, and β5 integrin shRNA was delivered into CD90 transfectant, respectively. The integrin shRNA-induced knockdown efficacy in HepG2 and Hep3B CD90 transfectant was analyzed by quantitative RT-PCR (Supplementary Figure S5A). The CD133 expression and anchorage-independent growth ability were not consistently inhibited by αV, β1, and β5 integrin shRNA (Supplementary Figure S5B and S5C). Given the effects of RLD domain of CD90 in CD133 expression and the colocalization of CD90 and β3 integrin [42], we next determined whether β3 integrin contributes to the CD90-induced phenotype in HepG2 and Hep3B cells. We delivered CD90 transfectants with integrin shRNA to silence β3 integrin (Figure 6A), and determined the expression level of CD133 and anchorage-independent growth. The expression of CD133 was reduced by β3 integrin shRNA (Figure 6B). CD90-induced phosphorylation of mTOR and AMPK were attenuated after the inhibition of β3 integrin (Figure 6C). Anchorage-independent growth was decreased in a similar fashion (Figure 6D). Anti-β3 integrin antibody effectively decreased the anchorage-independent growth induced by CD90 (Figure 6D). Co-incubation of tumor cells with lentiviral particles containing β3 integrin shRNA dramatically inhibited the tumor formation in mice (Figure 6E). In a therapeutic model, the lentiviral particles were delivered at 24 days after tumor implantation. Lentiviral expression of β3 integrinshRNA inhibited tumor growth (Supplementary Figure S6A). These data indicate that CD90 activates mTOR and AMPK to promote anchorage-independent growth via binding to β3 integrin.

## DISCUSSION

In this report, we demonstrate that the expression of the cancer stem cell marker CD90 enhances the tumorigenicity of liver cancer cells via the induction of CD133. Microenvironment carcinogenic stimuli, including HGF, TGF-β-1, and mutant HBV large surface protein, induce the expression of CD90 and CD133. The expression of CD133 is higher in CD90-positive cells than that in CD90-negative fresh tumor cells. Signaling analyses reveal that AMPK/mTOR and β3 integrin are required for the induction of CD133 by CD90. Importantly, the tumorigenicity of cancer cells can be blocked by shRNA or antibody targeting CD90, β3 integrin, and CD133. Finally, energy restriction mimetic agent OSU-CG5 effectively decreases the cells expressing CD90 and tumor growth. A scheme of signal axis of CD90 and CD133 and therapeutic opportunities is illustrated in Figure 6F. In accordance to our results, sorafenib is unable to induce apoptotic response on CD44+ cells in liver cancer [43]. This report suggests that therapy targeting signal axis of CD90 and CD133 may be combined with current therapy, such as sorafenib, in treating liver cancer.

Hepatic cancer stem cells have been identified by several markers [44, 45], including EpCAM, CD90, CD133, CD44, CD24, and CD13. CD133 is co-expressed with many other markers that are used to define liver cancer stem cells [23, 46]; thus, it is logical to hypothesize that there is a correlation between these markers. Our results demonstrated that ectopic expression of CD90 induced the expression of liver cancer stem cell markers, including CD133, CD24, and EpCAM. Our result provided the first evidence that expression of these stem cell markers are influenced by each other, which will be important for us to study the difference and lineage of cancer stem cells. For example, CD90 mainly induced the expression of CD133 and CD24, but had little effect on the expression of CD13. The correlation between these cancer stem cell markers provides a novel strategy to treat cancer. In this report, inhibitors targeting CD90-integrin-AMPK/mTOR-CD133 are effective in attenuating tumor progression. A recent study also indicates that CD24 contributes to the urothelial tumorigenesis and metastasis [47]. Therefore, targeting these cancer stem cell markers may be a general strategy for cancer treatment. CD90-positive cancer stem cell population is reported to have a characteristic of pro-glycolysis gene expression signature [48]. We have examined the therapeutic molecule OSU-CG5 which mainly targets glucose transporter and decreases energy sensing pathway. Sorafenib is better than OSU-CG5 in inhibiting cell growth. However, OSU-CG5 selectively decreased the CD90-positive cells which are potentially represented as cancer initiating cells. Therefore, combination of OSU-CG5 and sorafenib may provide a novel therapy for liver cancer.

Ectopic expression of mutant RLE CD90 did not affect mTOR phosphorylation, CD133 expression, and anchorage-independent growth. These results suggest that the binding of CD90 to integrin in *cis* is required for enhancing tumor formation. The *cis* binding of CD90 to integrin was also observed in the previous study describing the correlation between CD90 and ανβ5 integrin in myofibroblast. A binding of ανβ5 integrin to CD90 in *cis* competes the binding to latent TGF-β-1 complex, thereby inhibiting the contraction-iduced latent TGF-β-1 activation on myofibroblast differentiation and lung fibrosis [41]. On the other hand, CD90 binds to αvβ3 integrin *in trans* by syndecan-4 and leads to focal adhesion formation in melanoma invasion [49]. The tyrosine phosphorylation and focal adhesion formation in astrocyte are induced by thymoma expressing CD90 [50]. CD90 expressed on endothelium functions as a ligand for ανβ3 integrin expressed on melanoma and the interaction between ανβ3 integrin and CD90 in *trans* is correlated with the metastasis [27]. Furthermore, CD90 represents a counter receptor for leukocyte integrin Mac-1 and has the ability to bind the monocyte and PMNC (polymorphonuclear cell) [51]. In this report, a cis-interaction between CD90 and β3 integrin is required for anchorage-independent growth (Supplementary Figure S4). It will be important to study whether there is a trans-interaction between CD90 on liver cancer cell and the molecules on neighboring cells in hepatocellular carcinogenesis.

CD133 is expressed in many cancers, such as liver cancer, colon cancer, and brain gliomas [13, 14, 19]. Previous studies have also supported the notion that CD133 plays an oncogenic role in cancer. Overexpression of CD133 induces tumorigenicity in HEK293 cells [52], and CD133 is a potential oncogene in head and neck cancer [53]. The downregulation of CD133 by an HDAC6 inhibitor inhibits cancer cell differentiation [54]. Therefore, targeting CD133 and its related signaling pathways may be a feasible strategy for treating cancer.

In summary, our report indicates that the CD90-integrin-AMPK/mTOR-CD133 signal axis is required for hepatocarcinogenesis. We further provide evidences to support a new therapeutic approach by inhibiting the signal axis pathway between cancer stem cell markers.

## MATERIALS AND METHODS

### Reagents, chemicals and antibodies

Hepatocyte growth factor (HGF) and transforming growth factor β 1 (TGF-β-1) were purchased from Pepro Tech (Rocky Hill, USA). Rapamycin and AICAR were purchased from Calbiochem (San Diego, CA, USA). The anti-CD90 conjugated PE antibody was purchased from eBioscience (San Diego, CA, USA). The anti-EpCAM antibody was purchased from Santa Cruz Biotechnology (Santa Cruz, CA, USA). The anti-mTOR and anti-CD90 antibodies were purchased from Epitomics (Burlingame, CA, USA). The anti-CD133 and anti-CD44 antibodies were purchased from Abcam (Cambridge, UK). The anti-phospho-mTOR, anti-phosphor-AMPK, anti-AMPK, anti-mTOR, secondary horse anti-mouse horseradish peroxidase-conjugated and anti-rabbit horseradish peroxidase-conjugated antibodies were purchased from Cell Signaling (Beverly, MA, USA). The anti-β-actin antibody was purchased from Chemicon (Pittsburgh, PA, USA). The AC133 antibody was purchased from Miltenyi Biotec (Bergisch Gladbach, Germany).

### Cell lines

Hep3B, HepG2 and HuH7 cell lines were obtained from Dr. I.J. Su and Dr. T.M. Lin and authenticated by DNA (STR) profiling at Genelabs Life Science in Oct 2014 and Jan 2013, respectively. PLC/PFR/5 cell line was provided by B.C. Yang.

### Anchorage-independent growth ability

The protocol is modified from a previous report [36]. 1.5 ml of 0.6% agar in DMEM containing 10% FBS were prepared as underlayer in plastic petri dish. Five thousand cells to be tested were suspended in 1 ml of 0.3% agar in DMEM supplemented with 10% FBS and poured over the underlayer. The plates were placed in a 5% CO_2_ and 37°C atmosphere humidified incubator. The colonies were stained with 0.05% crystal violet after 14 days and photographed. The colonies were quantified using the Image-pro Plus software.

### Tumorigenicity in NOD/SCID mice

Male NOD/SCID mice were obtained from the Animal Center of the National Cheng Kung University (Tainan, Taiwan). The mouse experiments were approved by the Animal Welfare Committee at National Cheng Kung University. Five million HepG2, HuH7, HepG2 stable transfectants, or HuH7 stable transfectants were suspended in 200 μl of DMEM and implanted subcutaneously into the NOD/SCID mice. Ten million Hep3B and Hep3B stable transfectants were implanted into the NOD/SCID mice. To investigate the antitumor effects of sorafenib and OSU-CG5, sorafenib and OSU-CG5 were administered intraperitoneally with 10 mg/kg sorafenib or 50 mg/kg OSU-CG5 every other day after tumor implanted for 14 days.

### Flow cytometry

The trypsinized cells were labeled with anti-CD90-PerCp-Cy5.5, anti-CD133-PE, anti-CD45-APC, and anti-CD90-PE, respectively. After incubating with primary antibody, the cells were washed with phosphate buffered saline (PBS) and then resuspended in PBS supplemented with 1% FBS and 1 mM EDTA. The cells were analyzed using a FACS Calibur (BD. Biosciences, San Jose, CA, USA).

### Western blotting analysis

Cell lysate was prepared in RIPA lysis buffer. The protein concentration was determined with Micro BCA™ protein assay kit (Millipore, MA, USA). 35 μg protein was loaded into acrylamide gels and then transferred onto polyvinylidene fluoride membranes (Amersham Biosciences, Piscataway, NJ) after electrophoresis. The membranes were incubated with horseradish peroxidase-conjugated secondary antibody and probed with ECL western blotting detection system (Millipore, MA, USA) and visualized with the BioSpectrum AC imaging system.

### Sphere formation assay

Five thousand HepG2 and Hep3B stable transfectants were plated in the ultra-low attachment culture dishes (Corning Incorporated, Corning, NY, USA) in DMEM supplemented with 50 ng/ml HGF and 50 ng/ml EGF. The cells were incubated at 37°C, 5% CO_2_ for 14 days and then counted under light microscope.

### Quantitative real-time reverse transcription-PCR

Total RNA was extracted with TRIzol (MDBio, Taiwan). The cDNA was synthesized using M-MLV transcriptase (Promega, MI, USA). The quantitative PCR was performed using KAPA™ PROBE FAST qPCR Kit (KAPABIOSYSTEMS, Boston, Massachusetts, USA) on an Applied Biosystems StepOnePlus™ Real-Time PCR Systems. The CD44 and integrin β3 mRNA expression were analyzed using Maxima SYBR Green qPCR Master Mix kit (Fermentas, Canada). The CD90 primers were 5′-aggacgagggcacctacac-3′ (sense) and 5′-gccctcacacttgaccagtt-3′ (antisense); the CD133 primers were 5′-aaggcatatgaatccaaaattga-3′ (sense) and 5′-ccaccagag gcatcagaataa-3′ (antisense); the CD13 primers were 5′-ca tccatcagagatggcagac-3′ (sense) and 5′-tgctgaagagatcgtt ctgg-3′ (antisense); the CD24 primers were 5′-atgggcagagcaa tggtg-3′ (sense) and 5′-tggaataaatctgcgtgggta-3′ (antisense); the EpCAM primers were 5′-agttggtgcacaaaatactgtcat-3′ (sense) and 5′-ctcccaagttttgagccatt-3′ (antisense); the HPRT primers were 5′-tgatagatccattcctatgactgtaga-3′ (sense) and 5′-caagacattctttccagttaaagttg-3′ (antisense); the CD44 primers were 5′-tttgcattgcagtcaacagtc-3′ (sense) and 5′-gttacaccccaatcttcatgtccac-3′ (antisense) and the β3 integrin primers were 5′-ccgtgacgagattgagtca-3′ (sense) and 5′-aggatggactttccactagaa-3′ (antisense).

### RNA interference and lentivirus production

The shRNA targeting CD90, CD133, αV integrin, β1 integrin, β5 integrin and β3 integrin were obtained from National RNAi Core facility (Academia Sinica, Taipei, Taiwan). The shRNA used for silencing gene expression are following: TRCN0000057024, TRCN0000057025, TRCN0000062145, TRCN0000062146, TRCN0000010 768, TRCN0000010769, TRCN0000026945, TRCN00001 22920, TRCN0000057743, TRCN0000057744, TRCN 0000003236 and TRCN0000003237. The lentivirus production was managed according to the protocol provided from the National RNAi Core facility. For *in vivo* model with lentiviral shRNA, the CD90 transfectants and the lentiviral particles containing shRNA were co-injected subcutaneously into NOD/SCID mice. For therapeutic model with lentiviral shRNA, the HepG2 CD90 transfectants were implanted subcutaneously into NOD/SCID mice and the lentiviral particles containing luciferase or integrin β3 shRNA were delivered intratumorally at 24 days after transfectants implantation.

### Tissue samples

The tumor specimens were obtained from the National Cheng Kung University Hospital (Tainan, Taiwan) with the approval of the Institutional Review Board, and the tumor and non-tumor tissues were diagnosed by a pathologist. The specimens of hepatocellular carcinoma were obtained from Human Biobank within the Research Center of Clinical Medicine of the National Cheng Kung University Hospital (Tainan, Taiwan) with the approval of the Institutional Review Board (NCKUH IRB number: ER-97-148 and ER-101-245).

### Generation of CD90 RLE mutant cDNA

The RLD sequence of CD90 was mutated into RLE sequence with QuikChange™ site-directed mutagenesis kit (Stratagene, La Jolla, CA, USA) according to the manufacturer's instructions.

### Statistical analysis

All statistical analyses were performed using GraphPad Prism version 4 (GraphPad Software, La Jolla, CA, USA). The analysis was performed using unpaired *t*-test, one-way anova analysis and two-way anova analysis, respectively.

## SUPPLEMENTARY FIGURES



## Abbreviations

HCC: hepatocellular carcinoma
HGF: hepatocyte growth factor
TGF-β-1: transforming growth factor-β-1
CSC: cancer stem cell
