# Supplementary material for: Therapeutics targeting CD90-integrin-AMPK-CD133 signal axis in liver cancer
Source: Oncotarget. 2015 Oct 22;6(40):42923–37. doi: 10.18632/oncotarget.5976 (PMC4767481; doi:10.18632/oncotarget.5976)
Supplement: Supplementary file 1 [file oncotarget-06-42923-s001.pdf]

SUPPLEMENTARY FIGURES

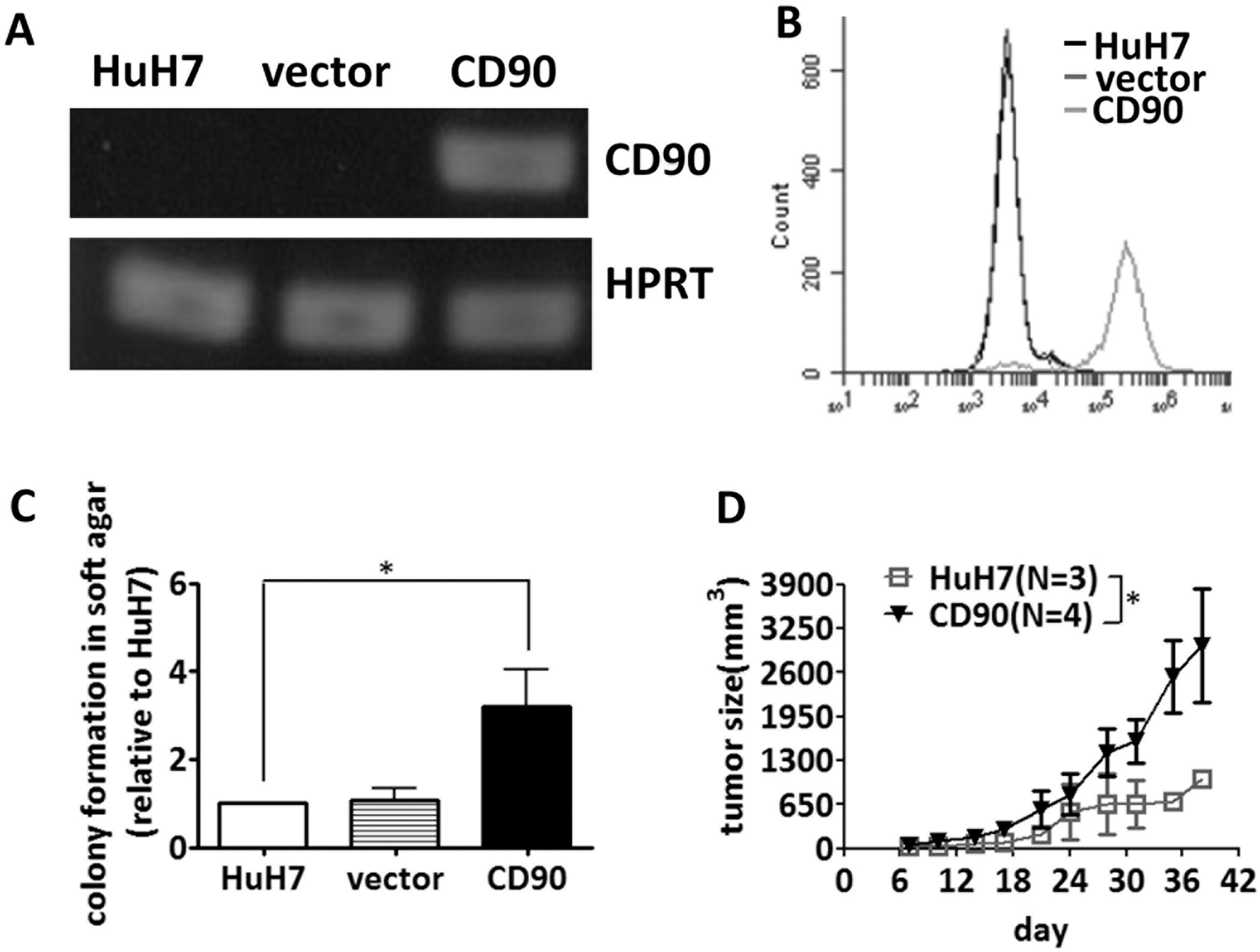

Supplementary Figure S1: CD90 promotes tumor formation in liver cancer HuH7 cell. CD90 mRNA expression and cell surface expression in the exogenous CD90-overexpressing stable HuH7 transfectants were determined by RT-PCR **A.** and FACS analyses **B. C.** The HuH7 transfectants were plated in soft agar, and the anchorage-independent colonies were monitored for 14 days. The colonies were quantified using Image-Pro Plus software. Data represent mean ± SEM ($n = 3$). P value was calculated using one-way anova analysis and * indicated $P < 0.05$. **D.** The HuH7 transfected cells were injected subcutaneously into NOD/SCID mice, and tumor growth curves were determined. Data represent mean ± SEM. P value was calculated using two-way anova analysis and * indicated $P < 0.0001$.

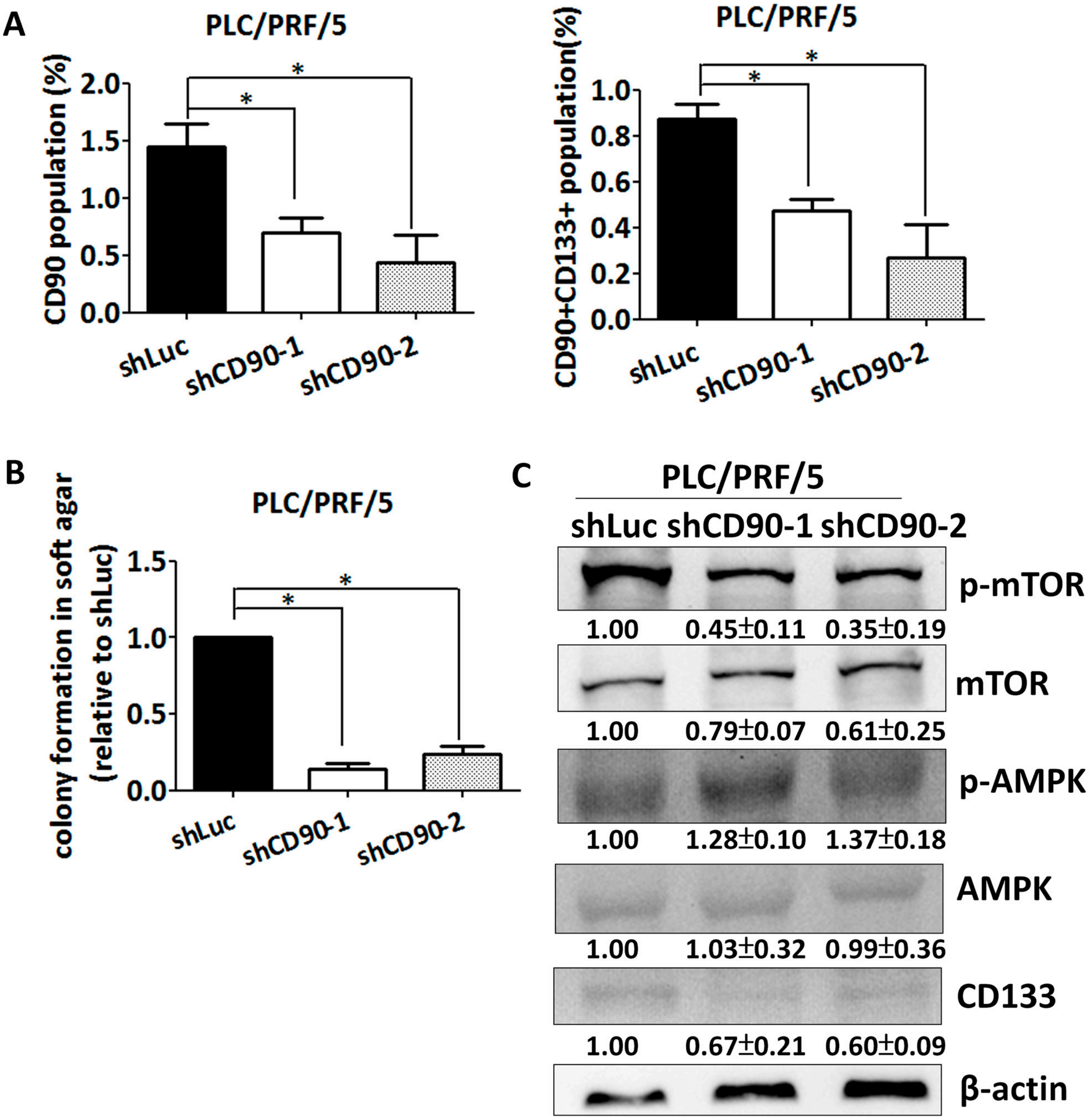

Supplementary Figure S2: Silencing of CD90 inhibits anchorage-independent growth and CD133 expression. PLC/PRF/5 cell was infected with lentiviral particles containing shCD90 or shLuc. **A.** The surface CD90 and CD133 in liver cancer PLC/PRF/5 cell was examined by flow cytometry. Data represent mean ± SEM (n = 4). **B.** Anchorage-independent growth was determined by soft agar assay after lentiviral infection of the CD90 shRNA. Data represent mean ± SEM (n = 4). P value was calculated using one-way anova analysis and * indicated $P < 0.05$. **C.** The phosphorylation of mTOR and AMPK in the PLC/PRF/5 cells was determined by western blotting. The quantitative data represent mean ± SD (n = 3).

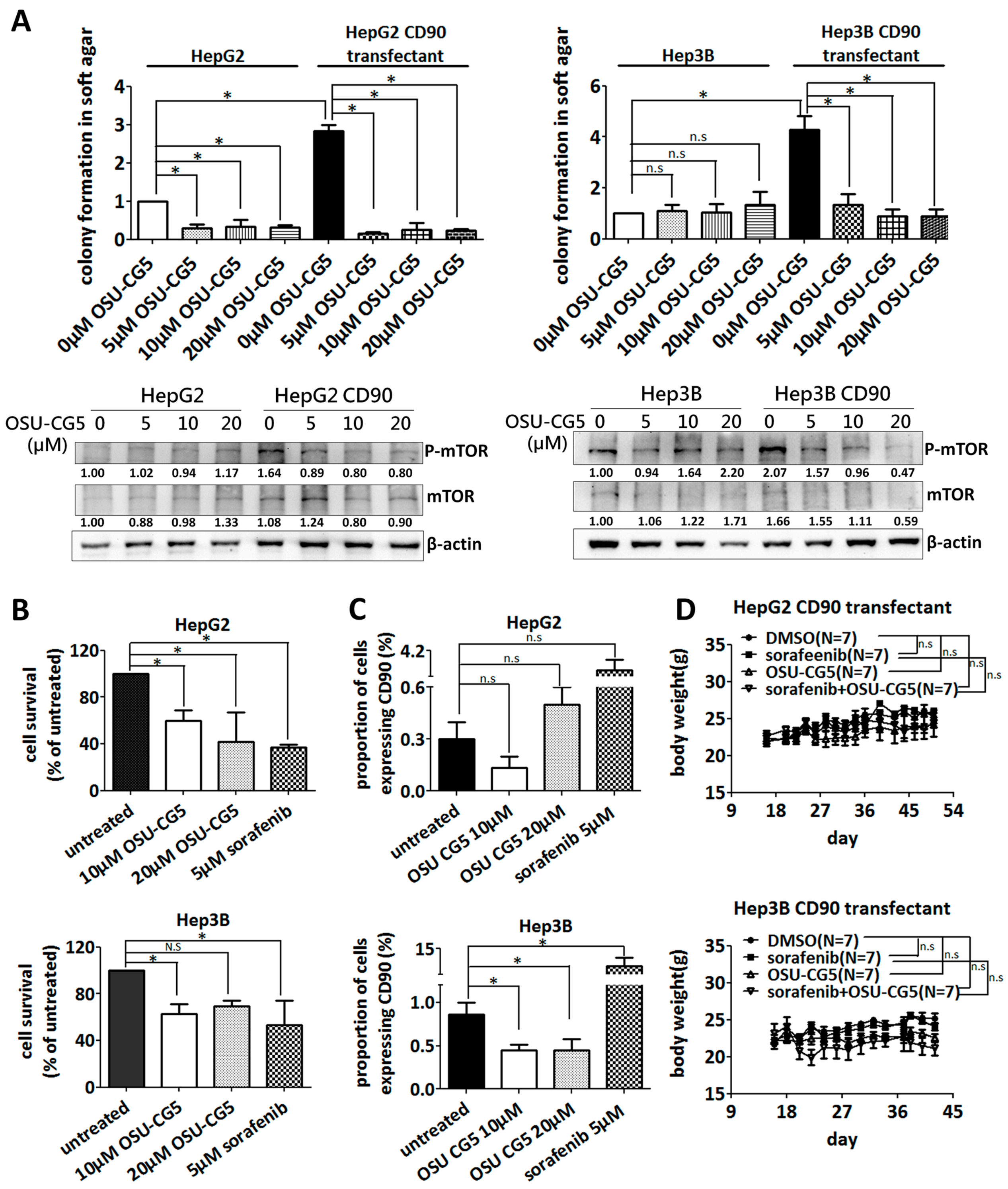

Supplementary Figure S3: Energy restriction mimetic agent, OSU-CG5, decreases the CD90 population in HepG2 and Hep3B cells. **A.** Upper panel, Anchorage-independent growth was determined using soft agar assay after indicated drug treatment. Data represent mean ± SEM (n = 3). Lower panel, the mTOR phosphorylatiom was determined by western blotting after treatment with the indicated drug. **B.** The number of the HepG2 and Hep3B cells was counted after OSU-CG5 or sorafenib treatment for 24 hours. Data represent mean ± SEM (n = 4). **C.** The CD90 population of the HepG2 and Hep3B was determined by flow cytometry 24 hr after the treatment with the indicated drug. Data represent mean ± SEM (n = 4). All P value was calculated using one-way anova analysis and * indicated $P < 0.05$. **D.** The body weight of the mice bearing CD90 transfectant with the indicated durg treatment was calculated. Data represent mean ± SEM. P value was calculated using two-way anova analysis.

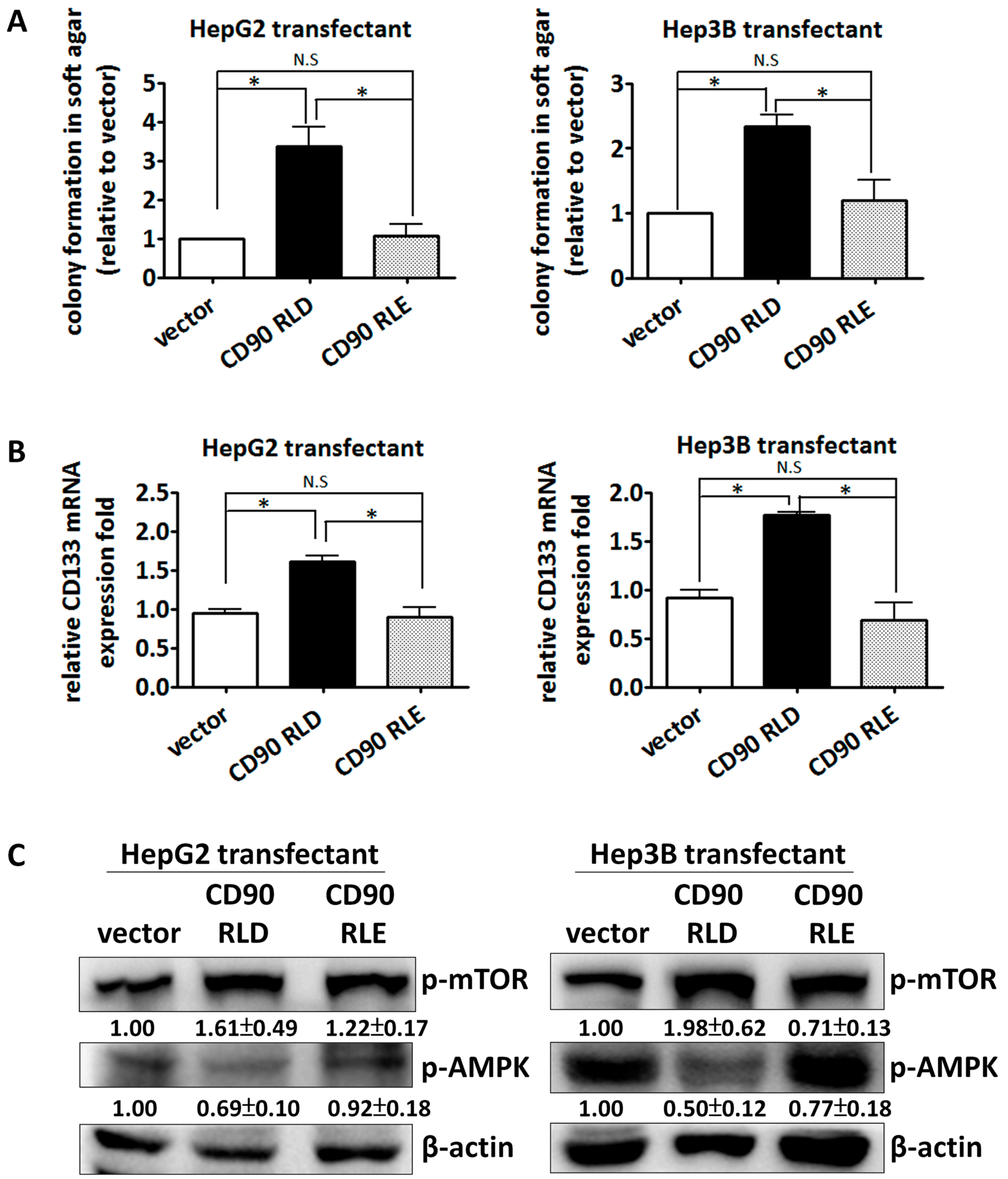

Supplementary Figure S4: The RLD domain of CD90 is essential for CD133 expression and anchorage-independent growth. A. Anchorage-independent growth and **B.** CD133 mRNA expression in the stable transfectants expressing wild-type CD90 (RLD) and mutant CD90 (RLE) were determined using soft agar assay and quantitative RT-PCR, respectively. Data represent mean ± SEM ($n = 3$). **C.** The phosphorylation of mTOR and AMPK in the transfectants expressing wild-type CD90 (RLD) and mutant CD90 (RLE) was determined by western blotting. Data represent mean ± SEM ($n = 4$). All P value was calculated using one-way anova analysis and * indicated $P < 0.05$.

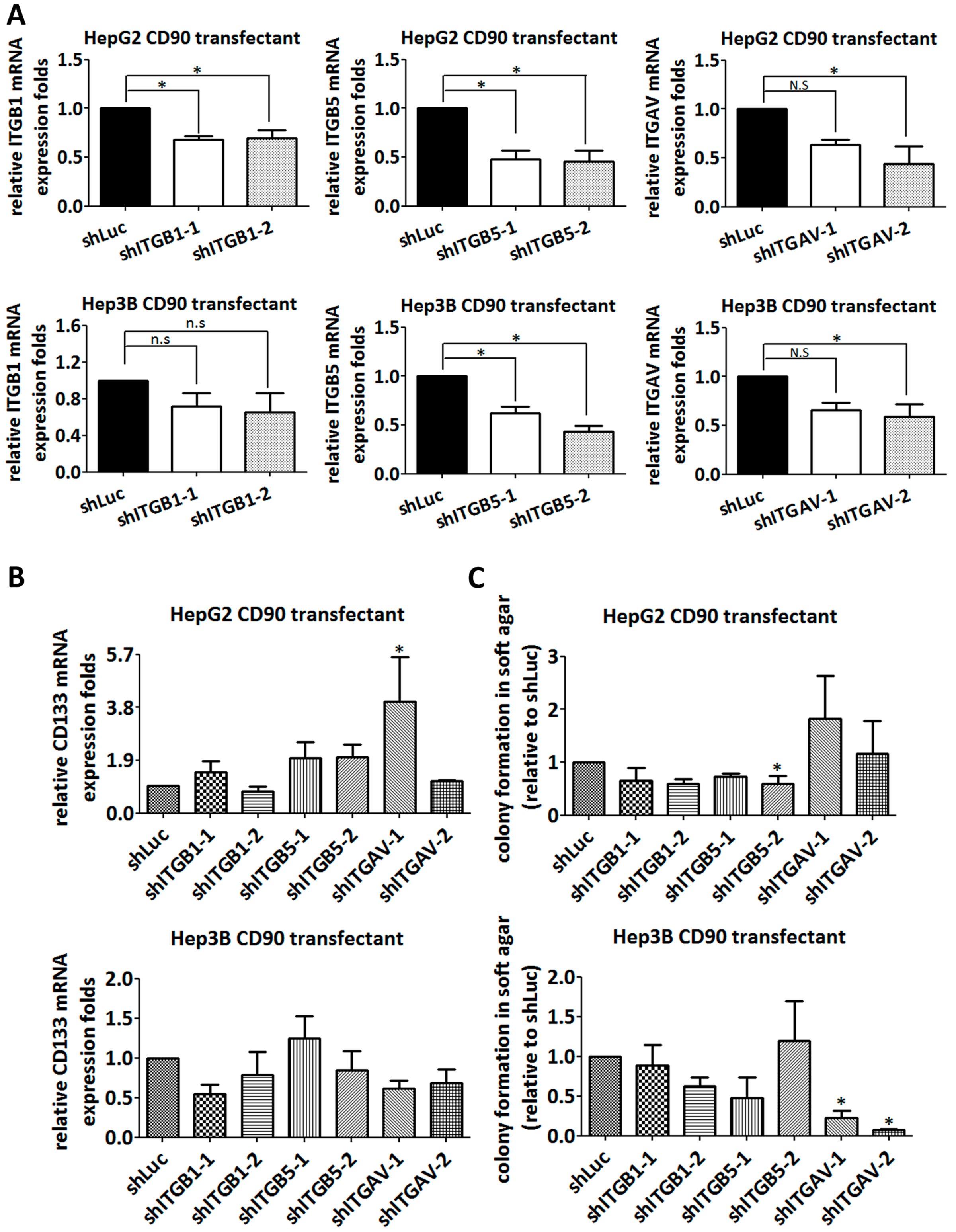

Supplementary Figure S5: The anchorage-independent growth induced by CD90 may not be caused by the expression member of integrin family, αV, β1 and β5. A. αV, β1 and β5 Integrin mRNA was downregulated in CD90 transfectants with lentiviral particles containing shRNA targeting αV, β1, β5 integrin and luciferase, respectively. **B.** CD133 mRNA expression in the cells expressing indicated shRNA was determined by quantitative RT-PCR. **C.** Anchorage-independent growth in the cells expressing indicated shRNA was determined by soft agar assay. Data represent mean ± SEM ($n = 4$). All P value was calculated using one-way anova analysis and * indicated $P < 0.05$.

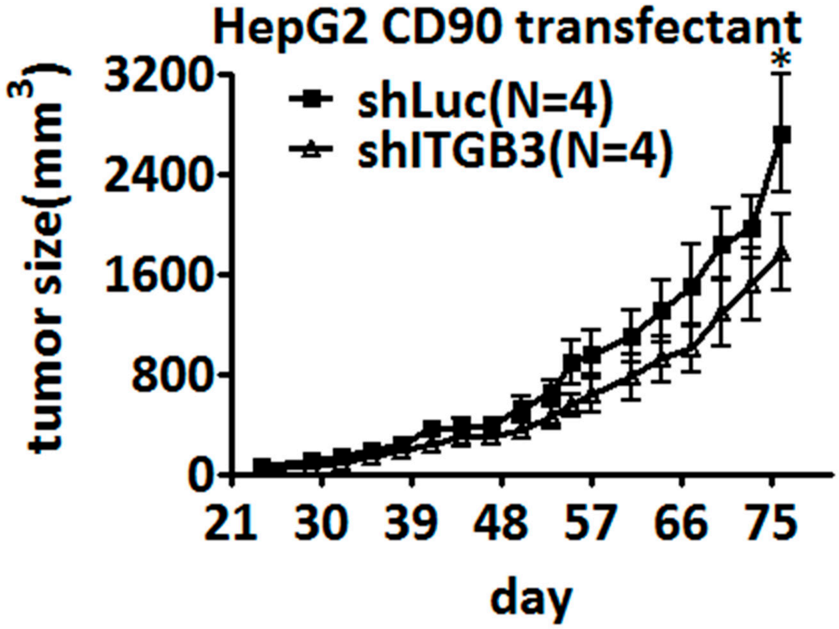

Supplementary Figure S6: β3 integrin shRNA inhibits tumor formation *in vivo*. HepG2 CD90 transfectants were implanted into NOD/SCID mice. After 24 days, the lentiviral particles containing luciferase or β3 integrin shRNA were injected intratumorally into the mice. The tumor growth was monitored twice a week. *P* value was calculated using two-way anova analysis and * indicated $P < 0.05$.
